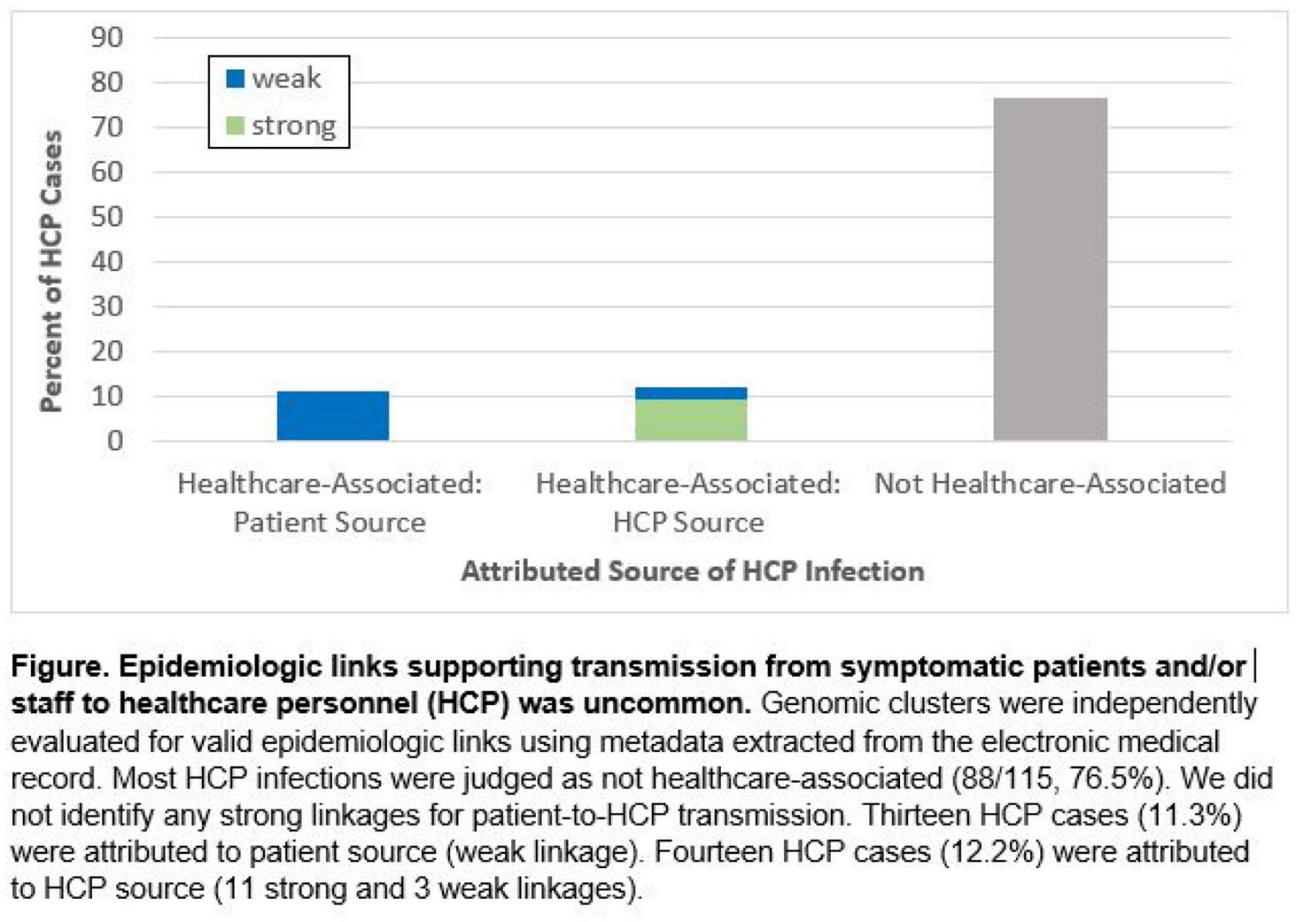# Genomic investigation to identify the source of SARS-CoV-2 infection among healthcare personnel

**DOI:** 10.1017/ash.2022.198

**Published:** 2022-05-16

**Authors:** Sarah Sansom, Hannah Barbian, Evan Snitkin, Christine Fukuda, Nicholas Moore, Lahari Thotapalli, Elias Baied, DO Young Kim, Mary Hayden, Michael Lin

## Abstract

**Background:** Contact tracing alone is often inadequate to determine the source of healthcare personnel (HCP) COVID-19 when SARS-CoV-2 is widespread in the community. We combined whole-genome sequencing (WGS) with traditional epidemiologic analysis to investigate the frequency with which patients or other HCP with symptomatic COVID-19 acted as the source of HCP infection at a large tertiary-care center early in the pandemic. **Methods:** Cohort samples were selected from patients and HCP with PCR-positive SARS-CoV-2 infection from a period with complete retention of samples (March 14, 2021–April 10, 2020) at Rush University Medical Center, a 664-bed hospital in Chicago, Illinois. During this period, testing was limited to symptomatic patients and HCP. Recommended respiratory equipment for HCP evolved under guidance, including a 19-day period when medical face masks were recommended for COVID-19 care except for aerosol-generating procedures. Viral RNA was extracted and sequenced (NovaSeq, Illumina) from remnant nasopharyngeal swab samples in M4RT viral transport medium. Genomes with >90% coverage underwent cluster detection using a 2 single-nucleotide variant genetic distance cutoff. Genomic clusters were independently evaluated for valid epidemiologic links by 2 infectious diseases physicians (with a third adjudicator) using metadata extracted from the electronic medical record and according to predetermined criteria (Table 1). **Results:** In total, 1,031 SARS-CoV-2 sequences were analyzed, identifying 49 genomic clusters with HCP (median, 8; range, 2–43 members per cluster; total, 268 patients and 115 HCP) (Fig. 1). Also, 20,190 flowsheet activities were documented for cohort HCP and patient interactions, including 686 instances in which a cohort HCP contributed to a cohort patient’s chart. Most HCP infections were considered not healthcare associated (88 of 115, 76.5%). We did not identify any strong linkages for patient-to-HCP transmission. Moreover, 13 HCP cases (11.3%) were attributed to patient source (weak linkage). Also, 14 HCP cases (12.2%) were attributed to HCP source (11 strong and 3 weak linkages). Weak linkages were due to lack of epidemiologic data for HCP location, particularly nonclinical staff (eg, an environmental service worker who lacked location documentation to rule out patient-specific contact). Agreement for epidemiologic linkage between the 2 evaluators was high (κ, 0.91). **Conclusions:** Using genomic and epidemiologic data, we found that most HCP COVID-19 infections were not healthcare associated. We found weak evidence to support symptomatic patient-to-HCP transmission of SARS-CoV-2 and stronger evidence for HCP-to-HCP transmission. Large genomic clusters without plausible epidemiologic links were identified, reflecting the limited utility of genomic surveillance alone to characterize chains of transmission of SARS-CoV-2 during extensive community spread.

**Funding:** None

**Disclosures:** None

**Table:**
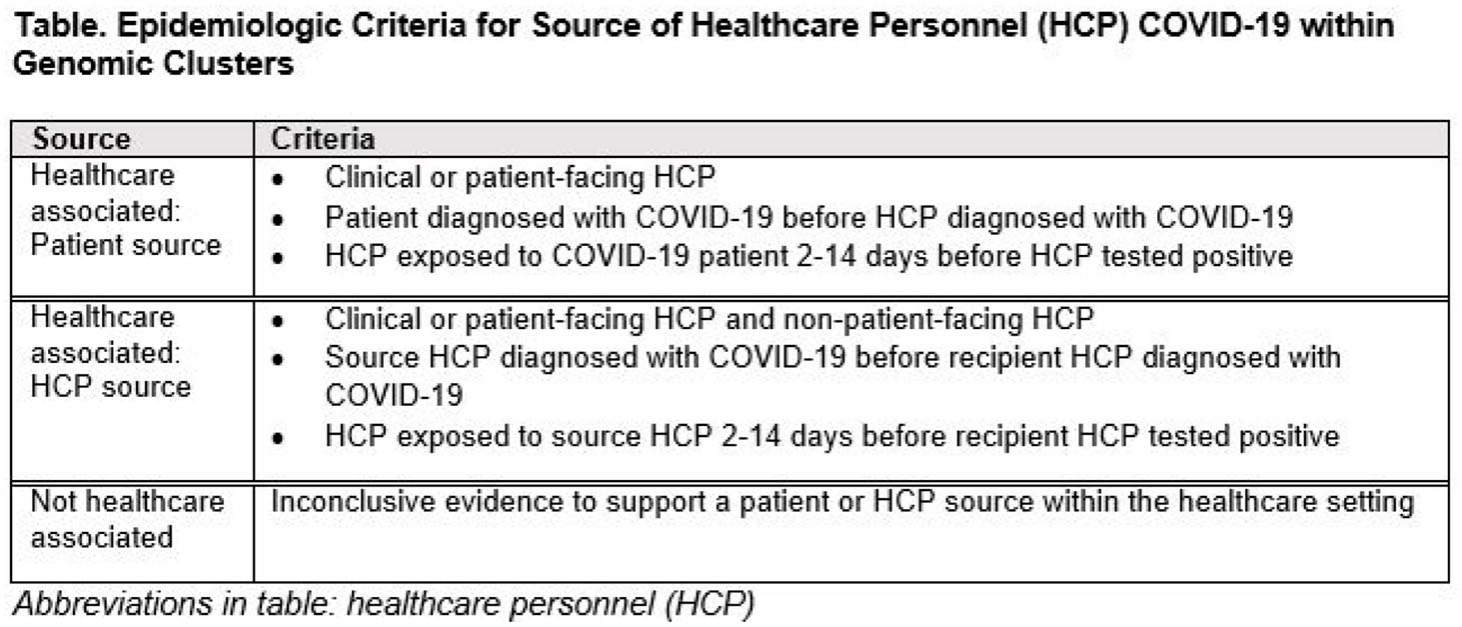

**Figure:**